# Estimation of Thermal Sensation Based on Wrist Skin Temperatures

**DOI:** 10.3390/s16040420

**Published:** 2016-03-23

**Authors:** Soo Young Sim, Myung Jun Koh, Kwang Min Joo, Seungwoo Noh, Sangyun Park, Youn Ho Kim, Kwang Suk Park

**Affiliations:** 1 Interdisciplinary Program for Bioengineering, College of Engineering, Seoul National University, Seoul 03080, Korea; kusashim@bmsil.snu.ac.kr (S.Y.S.); bioeng15@bmsil.snu.ac.kr (M.J.K); jkm6510@bmsil.snu.ac.kr (K.M.J.); 2 Samsung Advanced Institute of Technology, 130 Samsung-ro, Yeoungtong-gu, Suwon-si, Gyeonggi-do 16678, Korea; seungwoo.noh@samsung.com (S.N.); syun12.park@samgsung.com (S.P.); yh92.kim@samsung.com (Y.H.K.); 3 Department of Biomedical Engineering, College of Medicine, Seoul National University, Seoul 03080, Korea

**Keywords:** thermal sensation, thermal comfort, wrist skin temperature, wearable device

## Abstract

Thermal comfort is an essential environmental factor related to quality of life and work effectiveness. We assessed the feasibility of wrist skin temperature monitoring for estimating subjective thermal sensation. We invented a wrist band that simultaneously monitors skin temperatures from the wrist (*i.e.*, the radial artery and ulnar artery regions, and upper wrist) and the fingertip. Skin temperatures from eight healthy subjects were acquired while thermal sensation varied. To develop a thermal sensation estimation model, the mean skin temperature, temperature gradient, time differential of the temperatures, and average power of frequency band were calculated. A thermal sensation estimation model using temperatures of the fingertip and wrist showed the highest accuracy (mean root mean square error [RMSE]: 1.26 ± 0.31). An estimation model based on the three wrist skin temperatures showed a slightly better result to the model that used a single fingertip skin temperature (mean RMSE: 1.39 ± 0.18). When a personalized thermal sensation estimation model based on three wrist skin temperatures was used, the mean RMSE was 1.06 ± 0.29, and the correlation coefficient was 0.89. Thermal sensation estimation technology based on wrist skin temperatures, and combined with wearable devices may facilitate intelligent control of one’s thermal environment.

## 1. Introduction

In enclosed spaces such as an office, classroom, vehicle, and theater, several aspects of the environment provide comfort to people. Thermal comfort is expressed satisfaction with the surrounding thermal conditions, and it is a major condition among several comfort factors [1]. Maintaining thermal comfort improves quality of life and work effectiveness [2]. Specifically, an uncomfortable thermal condition can cause illness or inconveniences such as air conditioner sickness and waking up during sleep [3]. Previous studies have determined a relationship between a comfortable living environment and human productivity [4]. A previously reported approach for achieving thermal comfort is to configure the air conditioning system to the preferred temperature. However, as thermal comfort is subjective and multi-factor dependent, the optimal environmental temperature differs depending on an individual’s preference, clothing, activity level, *etc.* [5]. Therefore, an automatic system for controlling ambient temperature intelligently is required.

Thermal sensation is the sense of temperature between hot and cold, and it can be quantified using typical scales. For example, the numerical codes +3, +2, +1, 0, and −3 represent hot, warm, slightly warm, neutral, and cold, respectively, on the seven-point ASHRAE thermal sensation scale. Statistical analysis of the relationship between thermal sensation and thermal comfort showed that the comfort level was better than the onset of discomfort when thermal sensation votes (TSVs), based on nine-point analog scales, were between −1.5 and +2 [6]. As thermal sensation is related to thermal comfort, many researchers have developed a thermal sensation estimation model. As a general approach to thermal sensation estimation, the predicted mean vote (PMV) and standard effective temperature^*^ (SET^*^) should be considered [7,8]. The PMV model was designed to predict the average thermal sensation of a large population of people, and it was adopted as an ISO standard. The model considers six factors (four environmental factors, including air temperature, radiant temperature, air velocity, and relative air humidity, and two personal factors, including clothing and activity level) to assess thermal sensation from cold (−3) to hot (+3). SET^*^ indicates the equivalent air temperature of an environment at 50% relative humidity in which a subject, wearing clothing standardized for the activity concerned, has the same heat stress and thermoregulatory strain as in the actual environment [9]. However, these models reflect the average thermal response of a standard person [10]. As thermal sensation is subjective, a system that can assess individual thermal sensation is required.

To develop a model for personal thermal sensation estimation, many researchers have considered various physiological signals. Sugimoto proposed a wearable system that consisted of a tympanic temperature sensor, skin temperature sensors, and an electrocardiogram sensor with an accelerometer to evaluate individual thermal sensation [10]. The mean skin temperature calculated from skin temperatures measured from several parts of the body has been used to predict thermal sensation in various studies [11,12]. Moreover, as the number of arteriovenous anastomoses that is involved in thermoregulation is much greater in the hand than in other body surfaces, skin temperatures from the hand are often used in thermal sensation studies [6]. In a previously reported approach, the fingertip skin temperature combined with air temperature was proposed as a useful index for assessing individual TSVs [13]. Nakayama *et al.* developed an estimation algorithm that can estimate individual thermal sensation from the finger skin temperature, and they used the algorithm to control the household air conditioner [3]. In addition, the finger skin temperature or finger-forearm temperature gradient showed a high correlation (*r* = 0.78 and 0.80, respectively) with the overall thermal sensation [6]. Although thermal sensation estimation based on various physiological signals or the mean skin temperature showed remarkable outcomes, these approaches require multiple sensors across the whole body. Additionally, a sensor attachment in some parts of the body such as the forehead can be limiting in daily life. Also, even though the finger skin temperature is a useful physiological signal for thermal sensation estimation, it is necessary to determine how to attach a sensor around the finger for use in daily life.

Recently, a large number of wrist-type wearable devices that can monitor physiological signals have been released. Some wrist band devices such as the Basis Peak (Basis, San Francisco, CA, USA) and E4 wristband (Empatica Inc., Cambridge, MA, USA and Milan, Italy) provide wrist skin temperature monitoring. However, the feasibility of wrist skin temperature monitoring as a subjective thermal sensation estimation has not been sufficiently investigated. Although the analysis of data from a previous study indicated that the wrist would be the most responsive body location for evaluating thermal sensation, the estimation model based on wrist skin temperature was not provided and validated [14,15]. Therefore, in the present study, we investigated the applicability of wrist skin temperature monitoring to estimate subjective thermal sensation. Firstly, we invented a wrist-type wearable device that can simultaneously monitor skin temperatures from different parts of the wrist. Secondly, we developed an estimation model of individual thermal sensation based on wrist skin temperatures, and evaluated its performance. We expect that a smart environment system combined with wrist-type wearable devices will provide a suitable personalized environment in daily life. Moreover, this technology can contribute to energy conservation by preventing excessive energy consumption.

## 2. Experimental Methods

### 2.1. Wrist Skin Temperature Monitoring Device

**Figure 1:**
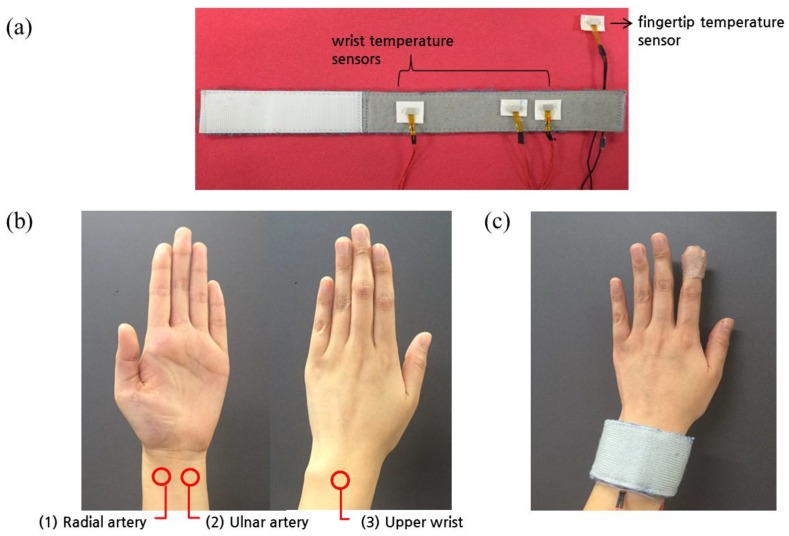
(**a**) A wrist band containing four temperature sensors; (**b**) The three wrist regions for skin temperature monitoring; (**c**) The wearable skin temperature monitoring device for the wrist and fingertip.

We have invented a wrist band that can simultaneously monitor skin temperatures from three different parts of the wrist—the radial artery (RA) region, ulnar artery (UA) region, and upper wrist (UW)—as shown in Figure 1. Firstly, the UW region is a valid candidate for skin temperature monitoring for wrist-watch type devices, as the main body of the device is usually located on the UW, and it is easy to embed the temperature sensor into it. Areas near the RA and UA were considered suitable, as they are the main arteries in the wrist, thus they would contain useful information on thermoregulation. The band was made from cotton, and the device included an additional temperature sensor that can monitor skin temperature from the fingertip. Fingertip skin temperature was acquired to evaluate the performance based on wrist skin temperatures compared to that from fingertip temperature.

**Figure 2:**
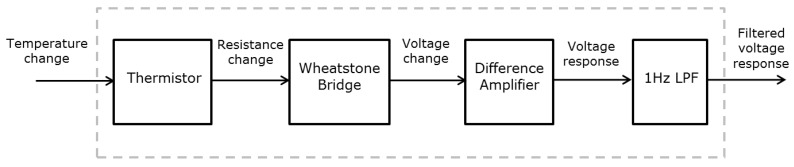
Temperature measurement circuit.

The temperature sensing module was designed with a protruding shape to ensure stable contact with the skin. A film-type NTC thermistor (LNJT103F, Lattron, Daejeon, Korea) was used, and each temperature sensor was covered with an thin aluminum cover. As each subject’s wrist size is different, we designed the temperature sensing module so that it could be moved and suitably positioned on each subject’s wrist region. The temperature measurement circuit was composed of a Wheatstone Bridge electrical circuit with a differential amplifier and a 1-Hz low-pass filter as shown in Figure 2.

After the sensing module and circuit were fabricated, each temperature sensor was calibrated for accurate temperature measurement. Sensor calibration was implemented using a stirred water bath, and we obtained the voltage output while heating the water from 19 °C to 41 °C. All sensors were attached to the copper plate floating on the water, and the surface temperature of the plate was measured using a wireless temperature sensing device (BN-SKT2, Biopac Systems Inc., Goleta, CA, USA). The relationship between the voltage output and temperature was curve fitted with a 4-order polynomial.

### 2.2. Experimental Protocol

**Figure 3:**
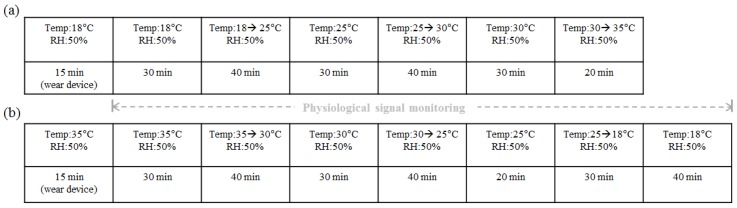
Two experimental protocols for ambient temperature control. (**a**) Ambient temperature increased from 18 °C to 35 °C; (**b**) Ambient temperature decreased from 35 °C to 18 °C. Temp and RH represent air temperature and relative humidity, respectively.

Eight healthy subjects (six men and two women, aged 26.63 ± 2.00 years, body mass index: 22.45 ± 2.63 kg/m^2^) participated in this study. Each subject wore trousers and a short-sleeve shirt (0.57 clo) [1]. To observe skin temperature changes with the thermal sensation variation, we adjusted the ambient temperature using a walk-in climate chamber (EBL-5HW2P3A-22, Tabai-Espec Corp., Osaka, Japan), according to the protocols shown in Figure 3. Skin temperatures were acquired from four subjects when the ambient temperature was increased from 18 °C to 35 °C. Then skin temperatures were obtained from the other four subjects when the ambient temperature was decreased from 35 °C to 18 °C. Relative humidity was maintained at 50% during the entire experimental sessions. The chamber was equipped with a ceiling that had hundreds of air holes, and the air was cooled or heated by a water circulation system. Air speed in the chamber was measured with an anemometer (Model A531, Kanomax Japan, Inc., Osaka, Japan) and maintained at < 0.2 m/s under a steady state condition. During the experiment, subjects sat on a chair in the center of the chamber and performed office work such as reading a document, using a smartphone, and writing. Subjects liberally moved their arms around or placed their arms on their leg while doing office work. To reduce the thermal effect of surrounding objects, direct contact with a table or wall was prohibited. The wrist band was worn on the subject’s non-dominant hand, and the fingertip temperature sensor was attached to the index finger. Skin temperatures from the fingertip, RA region, UA region, and UW were acquired every 1 s. The subjective perception of the overall thermal sensation was assessed using a 9-point scale: very hot (+4), hot (+3), warm (+2), slightly warm (+1), neutral (0), slightly cool (−1), cool (−2), cold (−3), and very cold (−4). Each subject was instructed to mark their subjective thermal sensation on a paper every 2 min to follow the variation in thermal sensation during experiment.

### 2.3. Development of the Thermal Sensation Estimation Model

As TSVs were surveyed every 2 min, skin temperature data were divided into 2-min segments. Then four parameters—the mean skin temperature (mSKT), time differential of the skin temperature (dSKT), temperature gradient between the measurement positions (gSKT), and average power of a specific frequency band (BP)—were calculated for each segment. The mSKT indicates the average skin temperature of a 2-min segment. As TSVs were surveyed every 2 min, the mean skin temperature was calculated based on 2-min segments. Also, dSKT was a useful predictor in various thermal sensation estimation models [11,16], and it was calculated as the difference of the mean skin temperature for the last 10 s compared to that of the initial 10 s. An analysis of data from previous studies indicated that the skin temperature (mSKT) and its time differential (dSKT) were closely related to the response of the cutaneous thermoreceptors and thermal sensation [17,18]. As altered skin temperature from the thermoneutral level can be a stimulus for thermal sensation [15], the skin temperature (mSKT), and its time differential (dSKT) were considered potential parameters for thermal sensation estimation. We varied the window from 1 s to 60 s to calculate the dSKT. As a result, the correlation coefficient between the dSKT and TSV differed only by 0.002 depending on the window. In this case, a short duration was usually preferred to benefit the calculation. However, we used the 10-s window to reduce the instantaneous noise in the skin temperature measurement. In addition, we considered the skin temperature gradients (gSKT) between the measurement parts, because the temperature gradients have served as a useful indicator in thermal sensation estimation in previous studies. For example, the temperature gradient from the fingertip to the forearm is a useful indicator for predicting vasoconstriction [19]. Additionally, the skin temperature gradient between the finger and forearm in conjunction with the finger temperature has served as a useful threshold for assessing discomfort from a cool environment [6]. The gSKT was obtained as the average value of the difference between the skin temperatures from two different measurement points. The time differential of the temperature gradient between measurement points (dSKT_g_) was also calculated. To comprehend the spectral characteristics, the BP was calculated. In a previous study, Nakayama *et al.* observed fluctuations in the peripheral skin temperature while one’s thermal sensation was between −2 and 2, and they surmised that the fluctuations resulted from the peripheral vessel’s vasomotion [3]. Therefore, we evaluated the applicability of spectral characteristics of wrist skin temperatures for thermal sensation estimation. Prior to the power calculation, the signal in each segment was smoothed with a 15-point moving average filter, and detrending was performed to focus on skin temperature fluctuations. Then the average power of four kinds of frequency bands—(1) from 0.05 Hz to 0.1 Hz; (2) from 0.1 Hz to 0.2 Hz; (3) from 0.2 Hz to 0.3 Hz; and (4) from 0.3 Hz to 0.5 Hz—was assessed. In each subject, the parameters of TSV were divided into two groups—a dataset for model development and a dataset for model validation.

To estimate thermal sensation, multiple linear regression analysis was performed. Specifically, stepwise regression was conducted to determine a useful subset of predictors and prevent overfitting. In each analysis, the criteria for entering and removing variables were 0.05 and 0.10, respectively. An estimation model based on a single skin temperature from the finger or one of the three wrist regions was developed. In addition, a model that uses multiple skin temperatures from the finger and various wrist regions was established to determine the effect of multi-sensor fusion. Finally, we analyzed whether the personal estimation model would improve the accuracy of the thermal sensation estimation. After the model was developed, each estimation model was assessed using a validation dataset. The mean root mean square error (RMSE), mean bias, and Pearson’s correlation coefficient were calculated to compare the performance of each estimation model.

## 3. Results and Discussion

During the experiment, 100,800 skin temperatures were acquired from each channel. Also, 840 TSVs were obtained—66 very hot (+4), 110 hot (+3), 159 warm (+2), 107 slightly warm (+1), 98 neutral (0), 86 slightly cool (−1), 52 cool (−2), 105 cold (−3), and 57 very cold (−4).

### 3.1. Relationship between the Skin Temperatures and Thermal Sensation

**Figure 4:**
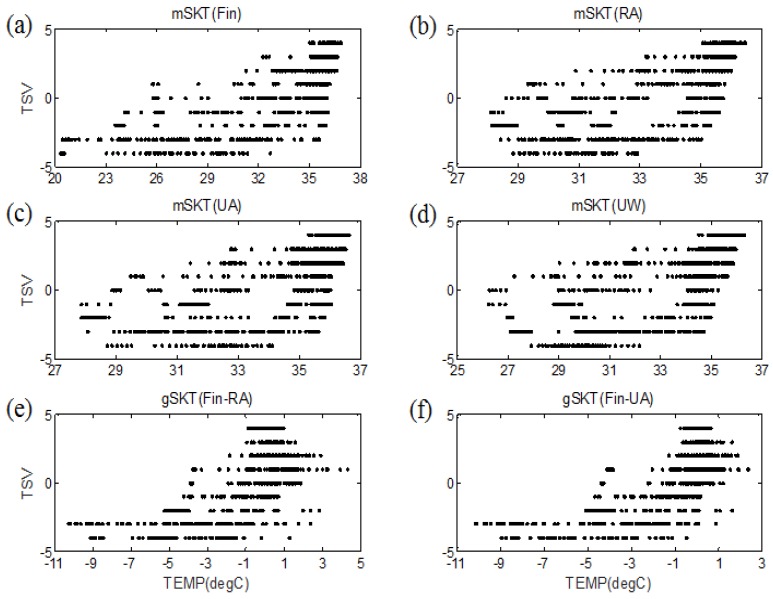
Relationship between the skin temperature parameters and thermal sensation votes (TSVs). Distribution of the mean skin temperature (mSKT) from the (**a**) fingertip; (**b**) radial artery (RA) region; (**c**) ulnar artery (UA) region; (**d**) upper wrist (UW); and the temperature gradient (gSKT) between the (**e**) fingertip and RA region; and (**f**) fingertip and UA region with respect to TSVs. Temp indicates temperature.

Before developing the estimation model, the relationship between the skin temperature and TSVs was determined. As shown in Figure 4a–d, the mSKT from each measurement position was positively correlated with TSVs. The mean skin temperature from the fingertip showed the highest correlation with TSVs (*r* = 0.73). Among skin temperatures from the wrist region, the mean skin temperature from the RA region and UW had correlation coefficients of 0.67 and 0.66, respectively. The correlation coefficient between the skin temperature measured at the UA region and TSVs was 0.61. In addition to a single channel observation, we evaluated the relationship between the gSKT and thermal sensation (Figure 4e–f). The gSKT between the fingertip and wrist had a stronger correlation with thermal sensation than that among the wrist regions. The gSKT between the fingertip and UA region had a correlation coefficient of 0.71. The gSKT between the fingertip and RA region, and UW had a correlation coefficient of 0.64 and 0.52, respectively, with respect to TSVs. Moreover, the temperature gradient among the three wrist regions had a relatively weak correlation with TSVs. The skin temperature gradient between the RA region and UA region or UW had an absolute correlation coefficient < 0.40. Also, the temperature gradient between the UA region and UW had a correlation coefficient of −0.50 with respect to thermal sensation. The dSKT from each wrist region had a higher positive correlation with the TSV than with the fingertip. However, the correlation coefficient between the dSKT and TSV was lower than 0.50.

**Figure 5:**
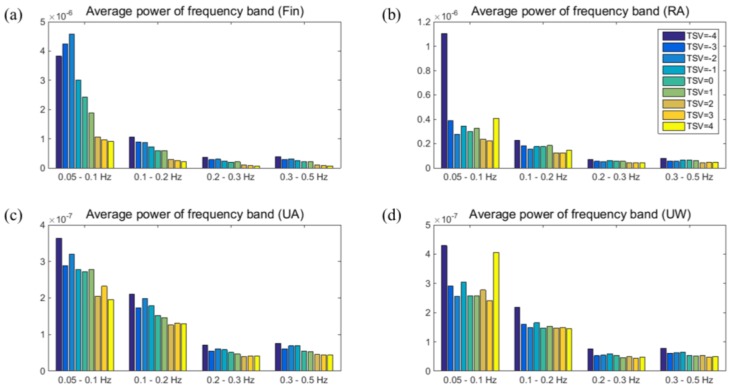
Relationship between the bandpower and thermal sensation votes (TSVs). Bandpower was calculated using skin temperatures measured from the: (**a**) fingertip (Fin); (**b**) radial artery (RA) region; (**c**) ulnar artery (UA) region; and (**d**) upper wrist (UW) region. Distribution of the mean bandpower in four frequency bands—from 0.05 Hz to 0.1 Hz, 0.1 Hz to 0.2 Hz, 0.2 Hz to 0.3 Hz, and 0.3 Hz to 0.5 Hz—is shown.

Although the mSKT or some gSKTs had a correlation higher than 0.60 in terms of thermal sensation, the range of mSKT was still broad in one scale of thermal sensation. Therefore, we included spectral parameters in the thermal sensation estimation model. As shown in Figure 5, the average power of each frequency band was generally decreased as the TSV increased. Specifically, there was a significant difference in the BP measured from the fingertip or RA when TSV = 1 (slightly warm) and TSV = 2 (warm) (α = 0.05). In addition, BP obtained from the UW was significantly different when TSV = −4 (very cold) and TSV = −3 (cold).

Some previous studies have reported a sex difference in thermal sensation [20,21]. The authors found that women were more sensitive to a temperature change from the optimal level than men. However, there was no distinct difference in various parameters depending on sex in this study. Additionally, we think that a larger number of subjects is needed to statistically analyze the effect of sex on thermophysiology.

### 3.2. Development of the Thermal Sensation Estimation Model

To develop the model, 422 datasets were used, and the rest of the 418 datasets were used to validate the model. We established four kinds of thermal sensation estimation models based on the: (1) single fingertip skin temperature; (2) single wrist skin temperature; (3) skin temperatures measured around the wrist; and (4) skin temperatures measured around the finger and wrist. Also, as we used a 9-point thermal sensation scale, the maximum and minimum estimated thermal sensation were limited to +4 and −4, respectively. Each estimation model is shown in Table 1.

To evaluate and compare the performance of each estimation model, we calculated the mean RMSE, mean bias, and correlation coefficient. Specifically, the mean RMSE and bias were respectively calculated based on the validation data of each subject and then averaged. As shown in Table 2, the thermal sensation estimation model using four skin temperatures (*i.e*., the fingertip and three wrist skin temperatures) showed the highest accuracy among the models. 

**Figure 6:**
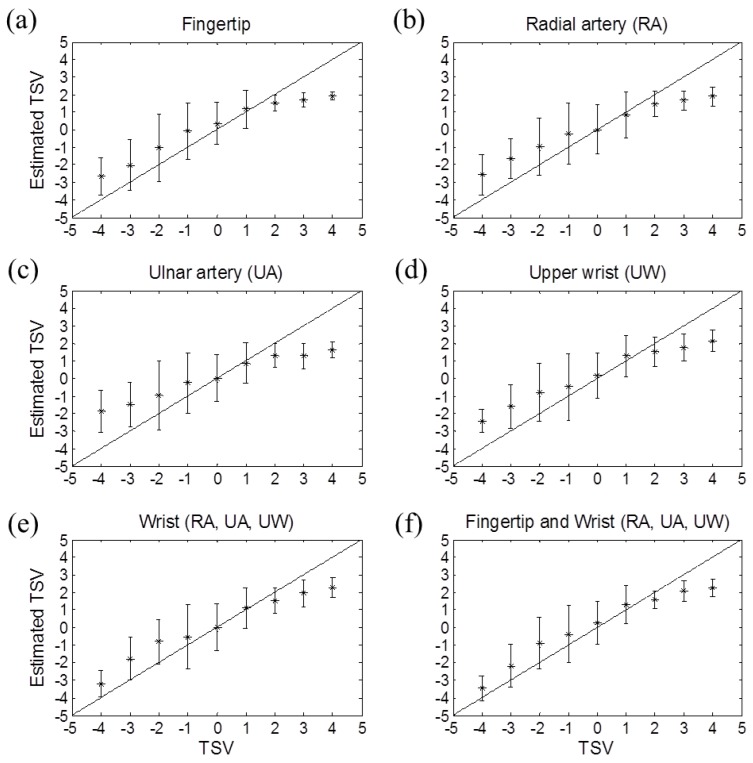
Measured *versus* the estimated overall thermal sensation in various measurement positions—(**a**) fingertip; (**b**) radial artery (RA) region; (**c**) ulnar artery (UA) region; (**d**) upper wrist (UW); (**e**) 3 wrist regions (RA, UA, UW); (**f**) fingertip and 3 wrist regions (RA, UA, UW). TSV means thermal sensation vote.

In addition, the estimation model based on three wrist skin temperatures showed a slightly better result to the model that used a single fingertip skin temperature. Among the models based on a single wrist skin temperature, the model with the RA or UW skin temperature provided a similar result. Thermal sensation estimation based on skin temperature from the UA region had the largest mean RMSE. Figure 6 shows the estimated TSVs of six estimation models.

**Figure 7:**
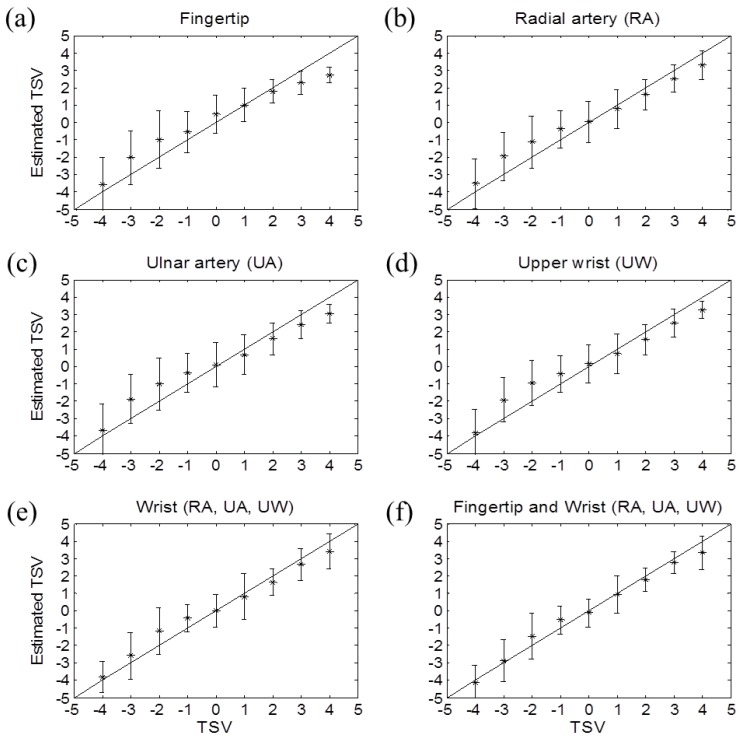
Measured *versus* the estimated overall thermal sensation in various measurement positions when using the personal estimation models—(**a**) fingertip; (**b**) radial artery (RA) region; (**c**) ulnar artery (UA) region; (**d**) upper wrist (UW); (**e**) 3 wrist regions (RA, UA, UW); (**f**) fingertip and 3 wrist regions (RA, UA, UW). TSV means thermal sensation vote.

After assessing the performance of six thermal sensation models, we established a personal thermal sensation estimation model, which was developed using individual data. As eight subjects participated in this study, each type of estimation model had eight different equations. Then each model was applied to the validation dataset of the corresponding subject. As shown in Table 3, the performance was improved; the mean RMSE decreased, and the correlation coefficient increased. The thermal sensation estimation model based on the fingertip and three wrist skin temperatures showed the highest accuracy (mean RMSE <1). Additionally, the model based on the three skin temperatures or the skin temperature near the UW had a smaller RMSE and a higher correlation than the model based on the fingertip skin temperature. Lastly, TSV was estimated without calculating spectral parameters when the model was based on skin temperature near the UA. In Figure 7, the estimated thermal sensation based on the personal model was close to the actual TSVs.

## 4. Conclusions

As various wrist-type wearable devices are available on the market, we evaluated the applicability of wrist skin temperature monitoring in thermal sensation estimation. Skin temperatures from three different parts of the wrist were monitored in sedentary people under various ambient temperatures, and the fingertip skin temperature was simultaneously measured. The highest accuracy was found using the estimation model based on the fingertip and three wrist skin temperatures. Also, the thermal sensation estimation model using the three wrist skin temperatures showed a slightly better performance to the model based on the fingertip skin temperature. When a personal estimation model was used, the accuracy of each model improved. In addition, the personal thermal sensation model based on the three wrist skin temperatures had a mean RMSE of 1.06 and a correlation coefficient of 0.89. Although a small group of subjects were included in the present study and the activity level was restricted to sedentary office work, we made an initial attempt to assess the feasibility of wrist skin temperatures for thermal sensation estimation and develop a specific estimation model based on wrist skin temperatures. Interest in thermal comfort is increasing with the desire to improve quality of life and energy conservation. Thus, wearable devices that can estimate thermal sensation may facilitate intelligent control of one’s thermal environment and provide personalized thermal comfort.

## Figures and Tables

**Table 1:** Various models for thermal sensation estimation *.

No.	Measurement Part	Estimation Model	Adjusted *R*^2^
**1**	**Fingertip**	TSV = 0.404 × mSKT + 0.918 × dSKT − 94,517.830 × BP_[0.05-0.1]_ − 387,622.240 × BP_[0.1-0.2]_ − 12.573	0.62
**2**	**Radial artery**	TSV = 0.670 × mSKT + 6.478 × dSKT − 4,009,175.078 × BP_[0.2-0.3]_ − 22.127	0.58
**3**	**Ulnar artery**	TSV = 0.644 × mSKT + 5.163 × dSKT − 4,051,375.063 × BP_[0.2-0.3]_ − 21.515	0.50
**4**	**Upper wrist**	TSV = 0.568 × mSKT + 6.414 × dSKT − 5,007,825.940 × BP_[0.3-0.5]_ − 18.159	0.61
**5**	**Radial artery, ulnar artery, and upper wrist**	TSV = 0.568 × mSKT_UW_ +1.048 × gSKT_(RA-UA)_+ 7.917 × dSKT_UW_ − 1.931 x dSKT_g(RA-UA)_ + 5.035 × dSKT_g(RA-UW)_ + 3,625,372.499 × BP_UA[0.2-0.3]_ − 5,033,252.737 × BP_UW[0.3-0.5]_ − 17.996	0.68
**6**	**Fingertip, radial artery, ulnar artery, and upper wrist**	TSV = 0.322 × mSKT_RA_ + 0.656 × gSKT_(RA-UA)_ − 0.681 × gSKT_(UA-UW)_ + 1.950 × dSKT_RA_ + 2.351 × dSKT_UA_ + 0.512 × dSKT_g(FIN-UA)_ − 74,000.163 × BP_FIN[0.05-0.1]_ − 238,485.732 × BP_FIN[0.1-0.2]_ + 4,154,333.783 × BP_UA[0.2-0.3]_ − 4,569,372.572 × BP_UW[0.3-0.5]_ − 8.970	0.72

* No., number; TSV, thermal sensation vote; mSKT, mean skin temperature; dSKT, time differential of the skin temperature; BP, average power of a specific frequency band; gSKT, temperature gradient between the measurement positions; dSKT_g_, time differential of the temperature gradient between measurement points.

**Table 2:** Performance of various thermal sensation estimation models *.

No.	Measurement part	Mean RMSE	Mean Bias	r
**1**	**Fingertip**	1.43 ± 0.46	0.00 ± 0.82	0.79
**2**	**Radial artery**	1.52 ± 0.33	−0.08 ± 0.82	0.76
**3**	**Ulnar artery**	1.70 ± 0.41	−0.12 ± 0.99	0.69
**4**	**Upper wrist**	1.53 ± 0.29	0.02 ± 0.83	0.76
**5**	**Radial artery, ulnar artery, and upper wrist**	1.39 ± 0.18	−0.05 ± 0.73	0.81
**6**	**Fingertip, radial artery, ulnar artery, and upper wrist**	1.26 ± 0.31	−0.01 ± 0.63	0.84

* No.**:** number; RMSE**:** root mean square error.

**Table 3:** Performance of the personal thermal sensation estimation models *.

No.	Measurement Part	Mean RMSE	Mean Bias	r
**1**	**Fingertip**	1.18 ± 0.48	0.09 ± 0.21	0.85
**2**	**Radial artery**	1.19 ± 0.38	0.06 ± 0.31	0.86
**3**	**Ulnar artery**	1.24 ± 0.39	0.04 ± 0.25	0.84
**4**	**Upper wrist**	1.16 ± 0.39	0.04 ± 0.21	0.86
**5**	**Radial artery, ulnar artery, and upper wrist**	1.06 ± 0.29	−0.01 ± 0.24	0.89
**6**	**Fingertip, radial artery, ulnar artery, and upper wrist**	0.95 ± 0.21	−0.06 ± 0.18	0.92

* No.**:** number; RMSE**:** root mean square error.

## References

[1] ASHRAE (2004). ANSI/ASHRAE Standard 55–2004: Thermal Environmental Conditions for Human Occupancy.

[2] Venneti K., Lenin M., Thilagavathy D.A. (2014). Ban technology based wearable wireless sensors for real-time environments. Appl. Mech. Mater..

[3] Nakayama K., Suzuki T., Kameyama K. Estimation of thermal sensation using human peripheral skin temperature. Proceedings of the 2009 IEEE International Conference on Systems, Man, and Cybernetics.

[4] Rawi M.I.M., Al-Anbuky A. (2013). Wireless sensor networks and human comfort index. Pers. Ubiquit Comput.

[5] Garcia-Souto M. (2012). Temperature and Comfort Monitoring Systems for Humans. Ph.D. Thesis.

[6] Wang D., Zhang H., Arens E., Huizenga C. (2007). Observations of upper-extremity skin temperature and corresponding overall-body thermal sensations and comfort. Build Environ..

[7] Fanger P.O. (1970). Thermal Comfort: Analysis and Applications in Environmental Engineering.

[8] Gagge A.P., Fobelets A.P., Berglund L.G. A standard predictive index of human response to the thermal environment. Proceedings of ASHRAE Transactions.

[9] Ye G., Yang C., Chen Y., Li Y. (2003). A new approach for measuring predicted mean vote (PMV) and standard effective temperature (SET∗). Build Environ..

[10] Sugimoto C. Human sensing using wearable wireless sensors for smart environments. Proceedings of 2013 Seventh International Conference on Sensing Technology (ICST).

[11] Takada S., Matsumoto S., Matsushita T. (2013). Prediction of whole-body thermal sensation in the non-steady state based on skin temperature. Build Environ..

[12] Yao Y., Lian Z., Liu W., Shen Q. (2007). Experimental study on skin temperature and thermal comfort of the human body in a recumbent posture under uniform thermal environments. Indoor Built Environ..

[13] Humphreys M.A., McCartney K.J., Nicol J., Raja I.A. An analysis of some observations of the finger temperature and thermal comfort of office workers. Proceedings of the 8th International Conference on Indoor Air Quality and Climate.

[14] Choi J.H., Loftness V. (2012). Investigation of human body skin temperatures as a bio-signal to indicate overall thermal sensations. Build Environ..

[15] Jacquot C.M., Schellen L., Kingma B.R., van Baak M.A., van Marken Lichtenbelt W.D. (2014). Influence of thermophysiology on thermal behavior: The essentials of categorization. Physiol. Behav..

[16] Zhang H., Arens E., Huizenga C., Han T. (2010). Thermal sensation and comfort models for non-uniform and transient environments: Part I: Local sensation of individual body parts. Build Environ..

[17] Hensel H. (1981). Thermoreception and temperature regulation. Monogr. Physiol. Soc..

[18] Ring J.W., de Dear R. (1991). Temperature transients: A model for heat diffusion through the skin, thermoreceptor response and thermal sensation. Indoor Air.

[19] House J.R., Tipton M.J. (2002). Using skin temperature gradients or skin heat flux measurements to determine thresholds of vasoconstriction and vasodilatation. Eur. J. Appl. Physiol..

[20] Schellen L., Loomans M.G., de Wit M.H., Olesen B.W., van Marken Lichtenbelt W.D. (2012). The influence of local effects on thermal sensation under non-uniform environmental conditions—Gender differences in thermophysiology, thermal comfort and productivity during convective and radiant cooling. Physiol. Behav..

[21] Karjalainen S. (2012). Thermal comfort and gender: A literature review. Indoor Air.

